# Stem Cell Therapy for the Treatment of Amyotrophic Lateral Sclerosis: Comparison of the Efficacy of Mesenchymal Stem Cells, Neural Stem Cells, and Induced Pluripotent Stem Cells

**DOI:** 10.3390/biomedicines13010035

**Published:** 2024-12-27

**Authors:** Lauren Frawley, Noam Tomer Taylor, Olivia Sivills, Ella McPhillamy, Timothy Duy To, Yibo Wu, Beek Yoke Chin, Chiew Yen Wong

**Affiliations:** 1 School of Medical, Indigenous and Health Sciences, University of Wollongong, Wollongong 2500, Australia; lauren.frawley123@gmail.com (L.F.); olivia.sivills@gmail.com (O.S.); emcphillamy@gmail.com (E.M.); 2 School of Biotechnology and Biomolecular Sciences, University of New South Wales, Sydney 2052, Australia; noam.taylor2@gmail.com (N.T.T.); t.to@blackdog.org.au (T.D.T.); wyb000512@gmail.com (Y.W.); 3 School of Health Sciences, IMU University, Kuala Lumpur 57000, Malaysia; 4 Center for Cancer & Stem Cell Research, Institute for Research, Development and Innovation (IRDI), IMU University, Kuala Lumpur 57000, Malaysia

**Keywords:** amyotrophic lateral sclerosis (ALS), induced pluripotent stem cells (iPSCs), mesenchymal stem cells (MSCs), neural stem cells (NSCs), regenerative medicine, stem cell therapy (SCT)

## Abstract

Background/Objectives: *Amyotrophic lateral sclerosis* (ALS), or Lou Gehrig’s disease, is a debilitating, incurable neurodegenerative disorder characterised by motor neuron death in the spinal cord, brainstem, and motor cortex. With an incidence rate of about 4.42 cases per 100,000 people annually, ALS severely impacts motor function and quality of life, causing progressive muscle atrophy, spasticity, paralysis, and eventually death. The cause of ALS is largely unknown, with 90% of cases being sporadic and 10% familial. Current research targets molecular mechanisms of inflammation, excitotoxicity, aggregation-prone proteins, and proteinopathy. Methods: This review evaluates the efficacy of three stem cell types in ALS treatment: mesenchymal stem cells (MSCs), neural stem cells (NSCs), and induced pluripotent stem cells (iPSCs). Results: MSCs, derived from various tissues, show neuroprotective and regenerative qualities, with clinical trials suggesting potential benefits but limited by small sample sizes and non-randomised designs. NSCs, isolated from the fetal spinal cord or brain, demonstrate promise in animal models but face functional integration and ethical challenges. iPSCs, created by reprogramming patient-specific somatic cells, offer a novel approach by potentially replacing or supporting neurons. iPSC therapy addresses ethical issues related to embryonic stem cells but encounters challenges regarding genotoxicity and epigenetic irregularities, somatic cell sources, privacy concerns, the need for extensive clinical trials, and high reprogramming costs. Conclusions: This research is significant for advancing ALS treatment beyond symptomatic relief and modest survival extensions to actively modifying disease progression and improving patient outcomes. Successful stem cell therapies could lead to new ALS treatments, slowing motor function loss and reducing symptom severity.

## 1. Introduction

Amyotrophic lateral sclerosis (ALS), or Lou Gehrig’s disease, is a debilitating and incurable neurodegenerative condition [1,2,3,4]. ALS is characterised by the death of lower motor neurons in the bulb and anterior horns of the spinal cord and upper motor neurons in the motor cortex [1,2,3]. The progressive muscle atrophy and spasticity associated with ALS lead to loss of daily function, paralysis, and eventual death [1,2,3,4,5].

Three theories have been proposed to describe the relationship between cortical hyperexcitability and motor neuron dysfunction in ALS pathogenesis: the dying forward, the dying back, and the independent hypothesis [6]. The dying forward hypothesis suggests that cortical hyperexcitability is the cause of preferential wasting in ALS via anterograde glutamatergic activity, where corticomotor neurons connect to anterior horn cells to mediate neurodegeneration [7]. This is specifically observed in the split-hand plus phenomenon, where the thenar group of intrinsic hand muscles have greater weakness compared to the hypothenar muscles [6]. Concordance between handedness further illustrates the impact of cortical hyperexcitability, with the dominant hand in upper limb ALS more likely to be the site of disease onset [6]. For this hypothesis, the likely mechanisms are primary motor cortex loss of inhibition and increased excitation. This is assessed in clinical trials using threshold-tracking transcranial magnetic stimulation to measure the function of Betz cells and intracortical neuronal networks within the primary motor cortex, known as short-interval intracortical inhibition (SICI) [6]. SICI acts as a biomarker for cortical inhibitory interneurons acting by γ-Aminobutyric acid type A (GABAA) receptor circuit function; the reduction or absence of SICI was noted to be a feature of sporadic ALS, linked to peripheral neurodegeneration [7]. In contrast, the dying back hypothesis argues that ALS begins at the neuromuscular junctions or the axon terminals of lower motor neurons, with the pathology then progressing towards the spinal cord and brain [8]. This hypothesis aligns with the early manifestation of muscle weakness and atrophy in ALS, suggesting an initial impact on lower motor neurons [6]. Lastly, the independent hypothesis suggests that upper and lower motor neuron degenerations occur in parallel but through separate pathological processes. This hypothesis accounts for the variability and complexity in ALS progression and symptomatology, acknowledging that both neuron types may have unique vulnerabilities and degenerative mechanisms.

The exact aetiology of ALS is unknown [1,2,5,9]. ALS is a disease of infrequent global incidence, with approximately 4.42 cases per 100,000 annually [1,10]. Despite genome-wide association studies estimating heritability at 52% and identifying 690 genes associated with ALS, only about 10% of ALS cases are familial, with the remaining 90% being sporadic [2,5]. Some of the main proposed mechanisms of motor neuron apoptosis in ALS include inflammation, glutamate-induced excitotoxicity, free radical build-up from superoxide dismutase type 1 (SOD1) point mutations, repeat-associated non-ATG translation due to C9ORF72 mutations, TAR DNA-binding protein 43 (TDP-43) proteinopathy, and aggregation-prone proteins [1,2,5,9,11,12]. Hung et al. (2023) discovered that phosphatidylinositol-3-phosphate 5-kinase type III kinase inhibition activates autophagy, and the ubiquitin–proteasome independent protein clearance mechanism is a particularly promising avenue of research [9]. This clearance mechanism is exocytotic and significantly attenuates ALS phenotype in animal and cell models [9]. Riluzole, functioning as a glutamate antagonist to decrease motor neuron excitotoxicity, is the only treatment proven to extend survival in ALS patients, typically by 2–3 months [1]. Tofersen is a 2023 Food and Drug Administration (FDA) approved drug designed to treat SOD1-ALS [13]. It functions as an antisense oligonucleotide that mediates the degradation of SOD1 messenger RNA, reducing SOD1 protein synthesis. The general safety of Tofersen has been demonstrated in a phase I/II clinical trial. However, a more extensive phase III trial identified that after 28 weeks, the primary endpoint for decreasing disease progression was unmet, even though a 35% reduction in SOD1 protein levels was noted in the cerebrospinal fluid [14]. Proposed to be completed in 2027, a separate phase III trial is underway to determine Tofersen’s impact on pre to early symptomatic patients [15]. Originally used to treat stroke patients, Edaravone was FDA-approved in 2022 as an ALS drug that scavenges free radicals with a phase III trial proposing greater baseline functionality but only for very early symptomatic patients with a forced vital capacity (FVC) >80% [16]. Also approved in 2022, sodium phenylbutyrate and taurursodiol targets both the endoplasmic reticulum and mitochondria of motor neurons to prevent cell death, with the CENTAUR 2022 trial reporting an average extended survival of 6.5 months [17]. In order to diagnose and understand disease progression, analysing ALS-related biomarkers in cerebrospinal fluid, blood, and other biological samples is essential to evaluate the impact of treatments. Biomarkers such as neurofilament light (NfL) and glial fibrillary acidic protein (GFAP) are particularly valuable for tracking neurodegeneration and glial activation, which are central to ALS pathology [18]. Increased levels of NfL in CSF and blood are closely linked to axonal damage and disease advancement, while GFAP levels indicate astrocytic activity and inflammation in the nervous system [19,20]. Similarly, inflammatory markers like C-reactive protein and interleukins (e.g., IL-6 and IL-10) provide insight into immune responses, both systemic and localised, which can be influenced by therapeutic interventions [21]. Given the multifaceted aetiology and significant heterogeneity in clinical presentations of ALS, targeting one molecular mechanism in treatment is unlikely to lead to a cure [1,5,7]. Consequently, stem cell therapy is being investigated as a multi-target approach to treating ALS [6].

Stem cells are unspecialised precursor cells, capable of both symmetrical division, producing clones, and asymmetrical division, generating differentiated daughter cells with the potential for multiple lineage specialisations [22]. Their fate and function are determined by the complex arrangement of epigenetic factors, including DNA methylation [23]. The ‘stem cell niche’ is formed by the interaction of surrounding cell types and their specific physiological environments, where transcriptional regulators and signalling cascades contribute to the determination of cell type [24,25]. Thus, depending on the microenvironment, these cells have a significant role in developmental and restorative processes across neonatal and adult stages of life, emphasising their essential status and importance in regenerative medicine [26].

Stem cells initially emerged in regenerative medicine with the first bone marrow transplantation by Georges Mathé in 1956 [27]. Their application has since expanded to treat various neurological disorders, including ALS. In 2015, South Korean company ‘CORESTEM’ introduced the first licensed ALS stem cell therapy, employing autologous intrathecal injections of mesenchymal stem cells (MSCs) derived from bone marrow stem cells (BMSCs) [28]. Recent studies have demonstrated the effectiveness of multiple stem cell types in analogous therapies. To assess how well transplanted stem cells integrate into neural circuits, markers like microtubule-associated protein 2 (MAP2) are used to reflect neuronal maturation and dendritic growth [29]. Safety is also paramount, and biomarkers such as p53 and c-Myc play a critical role in ensuring the genetic stability of stem cell therapies. While p53 serves as a safeguard against genomic instability, abnormal levels of c-Myc can indicate risks of tumour formation or undesired reprogramming [30,31]. Regular long-term biomarker tracking after treatment offers a means to evaluate the therapeutic effects to understand the durability of therapeutic outcomes, their influence on slowing disease progression, and the potential for any side effects.

**Figure 1:**
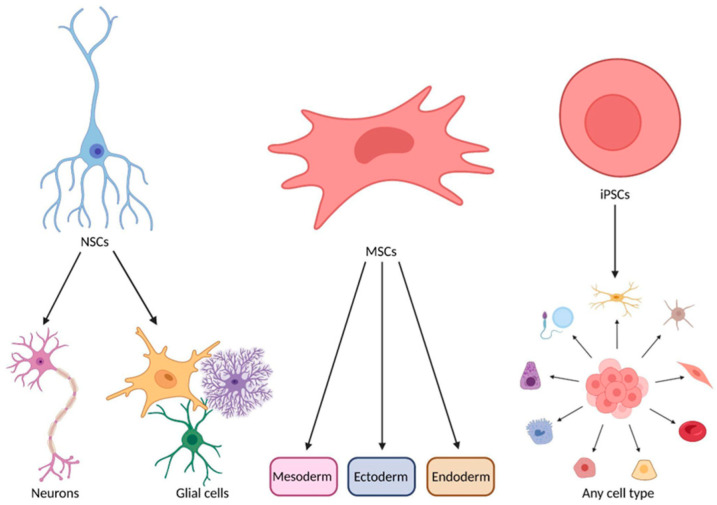
The differentiation potential of NSCs, MSCs, and iPSCs. NSCs differentiate into neurons and glial cells. MSCs have the potential to become cell types from the three germ layers: mesoderm, ectoderm, and endoderm. iPSCs can differentiate into any cell type. This figure was created using BioRender.

This review will compare three stem cell types which have either specific differentiation properties, immune modulation, or neuroprotective factors relevant to ALS’s neurodegenerative nature to assess their treatment efficacy: neural stem cells (NSCs), MSCs, and induced pluripotent stem cells (iPSCs). Stem cell potency, defined as the potential to differentiate into various cell lineages, is categorised as totipotent, pluripotent, or unipotent. This review investigates MSCs, which exhibit the ability to differentiate across mesoderm, endoderm, and ectoderm lineages, including neuronal cells, and NSCs, multipotent cells capable of proliferating into neurons and glial cells (Figure 1) [32]. MSCs, obtained from bone marrow, umbilical cord, and adipose tissue, are the most extensively studied stem cells in ALS clinical trials (Figure 1). iPSCs, engineered pluripotent cells developed by Shinya Yamanaka in 2006, offer a solution to ethical concerns associated with NSCs and MSCs [33]. Commonly derived from skin or blood cells, iPSCs can differentiate into ALS-relevant neurons and glial cells (Figure 1).

The regenerative and protective capacity of stem cells follows a variety of potential therapeutic mechanisms within ALS-affected tissue. MSC-derived conditioned medium, particularly from umbilical cord MSCs (UCMSC-CM), improves ALS outcomes in animal models by attenuating astrogliosis and microglial activation while inhibiting their lipopolysaccharide-induced inflammatory responses to prevent neurotoxic astrocyte phenotypes [34]. BM-MSCs and other MSC types protect neurons by increasing anti-apoptotic gene expression and suppressing oxidative damage [35]. While NSCs have been attempted for neuronal replacement, NSCs and their release of neurotrophic factors, such as brain-derived neurotrophic factor (BDNF) and glial cell line-derived neurotrophic factor (GDNF), have been extensively studied with promising improvements in synaptic plasticity, neuronal survival, and stabilisation of dendritic spines [36,37]. For iPSCs, their differentiation potential offers versatile mechanisms in ALS therapy, from direct replacement of motor neurons, astrocytes, and oligodendrocytes to restore neural networks and improve synaptic connectivity to modulating neuroinflammation by mitigating the neurotoxic effects of microglia and astrocytes [38,39]. However, despite ongoing research, there remains disagreement on the relative efficacy of NSCs, MSCs, and iPSCs in neurological disorders and their practicality in ALS treatment. Hence, we aim to evaluate the safety, efficacy, and prospective applications of stem cell therapy in ALS treatment by comparing NSCs, MSCs, and iPSCs.

## 2. Review Methodology

**Figure 2:**
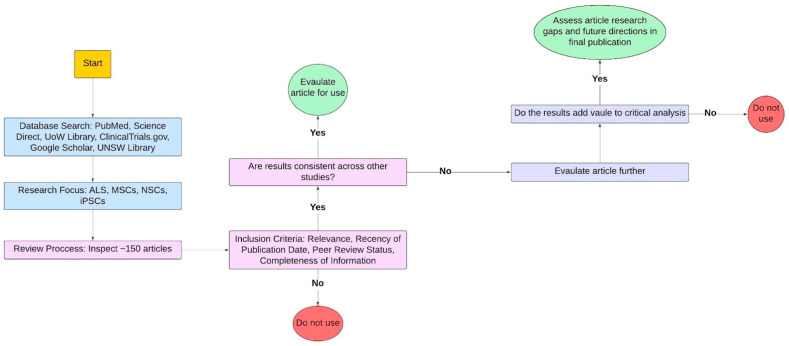
Methodological approach and criteria in determining source material suitability. Multiple online databases were used to inspect ~150 articles that were focused on ALS, MSCs, NSCs, and iPSCs. Studies that did not fulfil inclusion criteria were not used, whilst studies that did were assessed for consistency and evaluated. This figure was created with Lucidchart.

This study spanned from 23 December 2023 to 4 February 2024, utilising PubMed, ScienceDirect, University of Wollongong (UoW) Library, University of New South Wales (UNSW) Library, ClinicalTrials.gov, and Google Scholar databases. The study focused on key topics, including ALS and various types of MSCs, NSCs, and iPSCs (Figure 2). The review process involved inspecting approximately 150 articles, with selective inclusion criteria based on relevance, peer review status, and completeness of information (Figure 2). Outdated studies and those lacking peer review were excluded. BioRender and Lucidchart were employed in the construction of figures.

## 3. Mesenchymal Stem Cells in ALS Treatment

**Figure 3:**
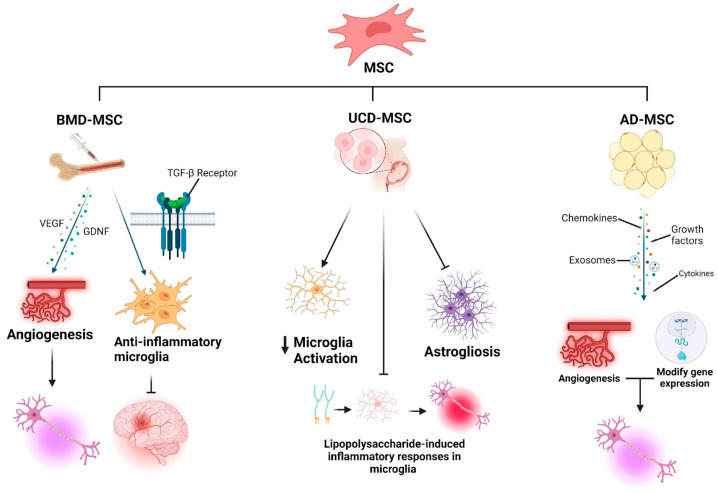
Mechanisms of MSCs in ALS Treatment. The figure illustrates the therapeutic pathways of BMD-MSCs, UCD-MSCs, and AD-MSCs in ALS. BMD-MSCs promote angiogenesis through the secretion of vascular endothelial growth factor (VEGF) and glial cell line-derived neurotrophic factor (GDNF), supporting neuronal survival and vascular remodelling. Additionally, BMD-MSCs engage in transforming growth factor-β (TGF-β) receptor signalling to induce anti-inflammatory microglia, which reduces neuroinflammation. UCD-MSCs are depicted as suppressing microglial activation and reducing astrogliosis, both critical contributors to ALS progression. Furthermore, UCD-MSCs attenuate lipopolysaccharide-induced inflammatory responses in microglia, mitigating neuroinflammation. AD-MSCs exert their effects through their secretome which is comprised of chemokines, growth factors, cytokines, and exosomes, which modulate gene expression, enhance angiogenesis, and promote neuronal repair. This figure was created using BioRender.

MSCs, distinguished by their capacity for self-renewal, potential for multi-lineage differentiation, and immunomodulatory characteristics, show promising therapeutic potential in treating ALS [40,41,42]. Derived from bone marrow, adipose tissue, and umbilical cord blood, MSCs exhibit neuroprotective and regenerative qualities [43]. Bone marrow-derived MSCs (BMD-MSCs) have been a focal point in ALS research [Table 1]. BMD-MSCs are known for their secretion of vascular endothelial growth factor and GDNF, which promote angiogenesis and motor neuron survival (Figure 3) [44]. Additionally, BM-MSCs release balanced levels of transforming growth factor-β, facilitating CNS homeostasis by shifting microglia from a pro-inflammatory to an anti-inflammatory phenotype (Figure 3) [44]. However, their proliferative capacity declines with donor age, making them less scalable for widespread applications [42]. A pivotal clinical study by Siwek et al. (2020) assessed intrathecal autologous BMD-MSC administration in ALS patients, involving three injections over three months [45]. The study found that while the safety of MSC injections was confirmed, the therapeutic effects varied among individuals. Using the ALS functional rating scale-revised (ALSFRS-R), a standardised tool used to evaluate the physical functional abilities of individuals with ALS, the study noted a slowdown in disease progression in late symptomatic patients [46]. However, in patients with a slower progression of ALS, there appear to be no significant changes in disease progression rates, indicating limited or no improvement from MSC therapy in these cases. The non-randomised and uncontrolled design and small sample size raise doubts about the causal relationships and extrapolative potential of the study [46]. In a parallel experimental study by Terashima et al. (2020), researchers combined bone marrow-derived mononuclear cells (BMD-MNCs) with growth factor-expressing MSCs in ALS mouse models [47]. The treatment significantly improved motor function, prolonged survival, and enhanced neuron preservation in ALS mouse models by promoting MNC migration and reducing gliosis, indicating an anti-inflammatory effect [47]. Furthermore, the treatment induced the expression of several cytokines in the spinal cord, suggesting an ability to modulate the immune response in ALS mouse models. The study had several limitations, including a small sample size, which restricted the ability to perform statistically significant analyses, inconsistency in the effects of treatment due to varying rates of disease progression among participants, and logistical challenges in managing treatment schedules for patients from distant locations, which affected adherence and follow-up. Furthermore, a retrospective study utilising intrathecal autologous BMD-MSCs (lenzumestrocel) compared against the pooled-resource open-access ALS clinical trials database showed improved survival probabilities with multiple injections, highlighting lenzumestrocel’s potential for enhancing long-term survival in ALS without significant adverse effects [28]. However, the retrospective nature of this study may indirectly impact the reliability of the data and necessitate larger, more rigorously controlled trials for a comprehensive evaluation of its long-term impact. Therefore, BMD-MSCs have been extensively studied in the context of ALS due to their ability to modulate immune responses, exhibit anti-inflammatory properties, and potentially contribute to neural repair. This offers a promising therapeutic strategy for managing ALS symptoms and progression [48].

Adipose tissue-derived mesenchymal stem cells (ASCs) have potential in ALS treatment due to their abundant availability, accessibility, high proliferation, and differentiation capabilities [49,50,51]. Research has highlighted the critical role of glial cells, particularly astrocytes and microglia, in neuroinflammatory responses and apoptosis, processes that are significantly implicated in the progression of ALS [52]. In regenerative medicine, the therapeutic potential of ASCs is accessed through their secretome, which comprises a diverse range of bioactive substances such as growth factors, cytokines, chemokines, and extracellular vesicles (Figure 3) [50]. The secretome plays a pivotal role in enhancing tissue repair by stimulating cell proliferation, facilitating angiogenesis, and exerting immunomodulatory effects. Specifically, ASCs offer therapeutic potential through the secretion of exosomes, small membrane-bound vesicles that are a part of the secretome [52]. These exosomes, filled with bioactive molecules including growth factors, can modify gene expression and influence recipient cell behaviour (Figure 3) [52]. Notably, an experimental study of transgenic mice models expressing the human SOD1 gene (G93A mutation) demonstrated that exosomes derived from stem cells (SCs) are effective in enhancing motor function, preserving lumbar motor neurons, neuromuscular junctions, and reducing glial cell activation [52]. The study highlights that intranasal administration of these exosomes presents a non-invasive, efficient delivery method directly targeting the CNS [52]. Furthermore, the study employed magnetic resonance imaging (MRI techniques using ultra-small superparamagnetic iron oxide nanoparticles to label ASC-exosomes, revealing their capacity to target CNS lesions specifically) [52]. This method highlights the potential of ASC-exosomes for precise delivery and modulation of the disease environment in ALS. This experimental study design offers significant advantages, including the ability to establish causal relationships between ASC-derived exosome administration and ALS outcomes due to the inclusion of a control group. Utilising a transgenic ALS mouse model enhances translational relevance, and the longitudinal design captures disease progression dynamics [52]. However, ethical considerations regarding animal welfare and the need for clinical validation in human ALS patients are essential limitations to acknowledge. Possible resolution of these issues may include the use of alternative models such as in vitro models (e.g., organoids) or computational modelling to predict disease mechanisms, progression, and treatment efficacy. This could reduce the reliance on animal models while focusing on more personalised, highly targeted treatment options by using patient-derived cells and data in low-risk environments. Parallel to experimental research, clinical trials play a critical role in assessing the safety and feasibility of MSC therapies in ALS. A trial at the Royan Institute and Mostafa Khomeini Hospital in Tehran assessed intravenously administered allogeneic adipose-derived MSCS in ALS patients [53]. Despite its limitations, the trial confirmed the safety of the treatment, though it did not conclusively prove its efficacy in altering disease progression. However, due to the absence of a control group and the trial’s open-label nature, there is difficulty in ascertaining the efficacy of the treatment relative to standard therapies or a placebo, with potential bias impacting subjective outcome measures. This underscores the necessity for more rigorously designed clinical trials to validate the effectiveness and safety of mesenchymal stem cell therapies in the context of ALS treatment. Therefore, ASCs show promise in ALS treatment through their rich secretome, which includes growth factors and cytokines that modulate neuroinflammatory responses crucial in ALS progression, highlighted by effective outcomes in experimental and clinical studies.

Recent advancements in the field of umbilical cord mesenchymal stem cells (UC-MSCs) have significantly contributed to the research on MSC therapies for ALS [34]. Tang et al. (2023) examined the efficacy of UC-MSC-derived conditioned medium (UC MSCS-CM) in ALS, which demonstrated that UCMSC-CM prolongs the lifespan of ALS model mice, attenuates microglial activation and astrogliosis, and diminishes inflammation [34]. UCMSC-CM was found to inhibit lipopolysaccharide-induced inflammatory responses in microglia and prevent the transformation of astrocytes into possessing a neurotoxic phenotype (Figure 3). These findings illustrate UCMSC-CM’s anti-inflammatory properties and potential as a clinical intervention for neuroinflammation in ALS. However, the extrapolation of these results to human ALS is constrained by the study’s reliance on animal models and the absence of corroborative human clinical data. Furthermore, the lack of a control group in the in vivo component of the research limits the breadth of generalisability regarding UCMSC-CM’s efficacy and safety in human ALS treatment. Wang et al. (2021) examined the genetic modification of human UC-MSCs-derived motor neurons through the transfection of the brain-derived neurotrophic factor (BDNF) gene [54]. Upon administering these modified cells to hSOD1G93A ALS model mice, they observed enhanced motor performance and prolonged survival, suggesting that integrating stem cell-derived motor neurons with BDNF overexpression could emerge as an innovative therapeutic approach for ALS. Complementing these findings, a retrospective case–control study involving 67 patients treated with Wharton’s jelly MSCs (WJ-MSCs) presented significant results [55]. Compared with a reference group from the PRO-ACT database, there was an observed doubling of the median survival time, highlighting WJ-MSC therapy’s safety and potential efficacy in certain ALS cases. This approach allows for the evaluation of treatment effects against a control group with similar baseline characteristics. However, its retrospective nature may limit data quality, and the absence of a randomised controlled trial framework restricts the findings’ ability to establish causality and generalisability.

A current phase III trial is being conducted utilising a multi-centre, randomised, double-blind, parallel-group, sham-procedure-controlled design (ALSUMMIT) to evaluate the efficacy and long-term safety of BMD-MSCs (lenzumestrocel) [56]. The results have yet to be published, making it difficult to assess the impact of the design on the findings and whether they address the aforementioned limitations of Nam et al. (2023) [28]. The study protocol of Nam et al. (2022) mentions a prior phase I/II trial, which demonstrated a notable therapeutic effect of BMD-MSCs persisting for a minimum of six months, along with safety for ALS patients [56,57]. Additionally, this trial observed a reverse relationship between TGF-β1 and MCP-1 levels following the injection. MCP-1 is an important biomarker in neuroinflammatory pathways that correlates with ALS progression and reduced survival [58]. The phase III trial completed by Cudkowicz et al. (2022) demonstrated statistically significant improvements in neuroinflammation when MSC-neurotrophic factor treatment was applied [58]. However, the trial notes that the participants were in advanced stages, which may have attenuated the treatment effect in the overall study population, as the primary and secondary efficacy endpoints were not statistically significant. Although this approach offers a more comprehensive representation by including a significant number of advanced ALS patients, it might have influenced the ALSFRS-R scale outcomes due to the floor effect. This effect suggests that any perceived improvements from low ALSFR-R scores could stem from misclassification of response, especially since the study’s aim was to demonstrate slowed progression.

**Table 1:** Summary of human clinical trials centring on MSC efficacy and safety.

Phase	Enrolment	Delivery	Dosage	Experimental Protocol	Reported Results	Reference
I	15	Lumbar procedure, intrathecal injection	Three doses at 3-month intervals	Autologous MSCs from patients’ bone marrow. Patients (aged 18–65). ALSFRS-R measured outcomes.	ALS patients with slow progression had no significant changes. Those with fast progression were slowed.	[45]
I/II	157	Intrathecal injection	Single cycle (2 injections), single cycle with boosters (3–10 injections)	Survival data of placebo participants from PROACT database. Propensity score matching. Multivariable Cox proportional analysis.	BM-MSC group had a higher survival probability and a lower hazard ratio for death. Multiple injections were more effective. No serious adverse drug reactions up to a year.	[28]
I/IIa	20	Intravenous infusion	2 × 10^6^ cells/kg	ALSFRS-R ≥ 24 FVC ≥ 40% Riluzole (100 mg), twice a day. Exclusion of comorbidities.	Two patients experienced dyspnea and chest pain caused by pulmonary emboli. Three deaths were recorded. Five patients survived at five-year follow-up.	[53]
III	196	Intrathecal injection	Three doses	El Escorial criteria. ALSFRS-R ≥ 25 (screening) and ≥3 ALSFRS-R points decline prior to randomisation. Participants (aged 18–60) randomised 1:1 to MSC-NTF:placebo.	No safety concerns to MSC-NTF. Significant improvements are cerebrospinal biomarkers of neuroinflammation, neurodegeneration, and neurotrophic factor support.	[58]
III	115 (estimated)	Lumbar puncture at L2–L4	Group 1 and 2 will have the lenzumestrocel injection, concentration depending on subject’s weight (1 × 10^6^ cells/kg). All groups will have 5 injections in total.	Combined assessment of function and survival: 6 months for Group 1 vs. control, 12 months for Group 2 vs. control after the first administration.	No reported results yet, and safety assessment is to occur during the 56-week main study that is expected to conclude in May 2026. Follow-up observational study will be conducted to evaluate long-term efficacy and safety for up to 36 months.	[56]

## 4. Neural Stem Cells in ALS Treatment

NSCs are multipotent, self-renewing cells isolated from the fetal spinal cord or brain that can differentiate into neurons, astrocytes, and oligodendrocytes [32,49]. As determined by Chen et al. (2018) in a quantitative gene expression study on NSC lineage, three subtypes can be identified: activated NCSs, quiescent neural stem cells, and neural progenitor cells, which all contribute to different developmental roles [59]. Sharing the exact tissue origin of degenerated cells and therefore being compliant with microenvironmental signals, NSCs have been a promising area of research in ALS therapy. However, as age is ALS’s main identifiable risk factor, the extrinsic and intrinsic regulation of adult neurogenesis is both an essential and limited area of research, with qNSCs predominantly reduced to specific adult brain regions and consequent ethical and physical isolation limitations [25,60]. Despite increasing knowledge surrounding adult neurogenesis, critical questions such as the functional integration and connection sourcing of new neurons still need to be addressed before the NSCs can become a viable treatment for any neurological disorder. This research focuses on adult NSCs, which would be more relevant to age-progression neurological diseases such as ALS and help to address consistent ethical and practical issues related to the isolation and use of fetal-derived NSCs. Common delivery procedures such as intraspinal injections and transplantation are technically demanding and raise concerns about patient safety, long-term efficacy, and the moral implications of using fetal tissue. These issues are further complicated by the fact that fetal-derived tissues are limited in availability, making large-scale or routine clinical applications unfeasible. Furthermore, the genetic modification of fetal or embryonic NSCs, which might be necessary to enhance their therapeutic potential, is often seen as non-compliant with ethical standards due to fears of unintended genetic consequences and potential misuse. Adult-derived NSCs can circumvent many of these ethical concerns and allow for personalised therapies that reduce risks of immune rejection and align more closely with regulatory and societal expectations. Multiple animal model trials [Table 2] have used SOD1G93A rodents as the main subjects to model disease progression and efficacy of human NSCs (hNSCs) with ALS [61]. These trials indicate a beneficial effect of intraspinal hNSC injection primarily in L1–L4 in pre/early symptomatic animal models. However, they face comparison issues related to the genetic background variability in mix-strained mice colonies and encounter mixed results concerning hNSC migration in surrounding tissue. Only partial differentiation was reported to occur in healthy glial cells, with less than 1% expressing either neuronal or astroglial markers (TUJ1 and GFAP, respectively) [62]. This limited proliferation and self-renewal ability may contribute to the minimal improvements in rodents with advanced disease progression [62,63]. Contrary to these results, Zalfa et al. (2019) found extensive hNSC migration (up to 3.77 ± 0.63 cm) along the rostral–caudal axis throughout the thoracic and sacral segments where partial differentiation was reported to correlate with an extended lifespan of approximately 30 days compared to the control [64]. Utilising similar methodologies to Teng et al. (2012), which had an increased sample size of data sourced from 11 independent studies, bilateral hNSC injections in the lumbar spinal cord suggest that grafting at multiple levels in the spinal cord reaches and protects broader neighbouring motor neurons [62]. Similarly, two-site transplantation successfully resulted in a 17-day increased lifespan, but future studies are needed to determine this effect at different disease progressions [65]. The recent Tzeplaeff et al. (2023) trial branched from previous studies to focus on hNSC survival length in various injection pathways, critical for more advanced ALS progressions and reducing cell rejection [66]. Although limited effects on survival time were noted, the lateral ventricle pathway had a more positive protein assay analysis on hNSC density over the tail vein pathway [66]. This supports the need for multiple grafts along the CNS with the lack of hNSC migration and proliferation, a potential concern for the complexity of human ALS phenotypes.

Building on the relative success in animal models, early-stage research and trials involving NSCs have primarily concentrated on the safety and tolerability of intraspinal hNSC injections. These studies yielded numerous promising results [Table 3], leading to FDA approval for such trials in 2009 [67]. Two phase I clinical trials have been conducted. The first, funded by the biopharmaceutical company Neuralstem, demonstrated the absence of long-term complications, with 12 patients monitored for up to 18 months after surgery [68]. Due to the success in all 12 patients, three additional patients received an additional five unilateral cervical injections in a phase II trial with no identified deceleration in motor neuron function [69,70]. The second phase I trial extended the analysis to 60 months, showing no negative effects or signs of iatrogenic damage [71]. As an identified problem in SOD1G93A rodent models, NSC’s lack of proliferation and migration limits its effect on broader symptoms and more advanced disease types [63]. Consequently, a trial at Cedars-Sinai Medical Center became the first to use genetically modified progenitor cells (CNS10-NPC-GDNF) to treat ALS. These cells are modified to contain glial cell-derived neurotrophic factor (GDNF) to specifically differentiate into astrocytes while supporting the survival of other neuronal cells [72]. Injected unilaterally to the lumbar region in 18 ALS patients with moderate leg movement, the stated primary safety endpoint of 12 months was met with no adverse effects on motor function up to 42 months [72]. However, due to the often rapid disease progression of ALS paired with low sample size potentials, a lack of methodology homogeneity meant that safety time thresholds were potentially too low to properly analyse long-term effects with rapidly deteriorating symptoms for already advanced phenotypes of available recruited patients. Consequently, deaths due to the natural progress of disease were observed even when these trials restricted participation to patients with an FVC ≥ 60% of the predicted value for both ambulatory and non-ambulatory groups [71,72,73]. Despite complications, the studies suggest relative safety, meeting safety time thresholds of at least 12 months and up to 60 months with varied deliveries and dosages [71,72,73]. This is supported by post-mortem tissue analysis, where 13 of the 18 patients dying in the Baloh study showed graft survival and GDNF production [72]. Extending past potential safety assessments, the trials also demonstrated positive results in potential efficacy. One patient demonstrated improvements in disease progression in motor neuron and rotarod performance tests, and another three recorded transient functional improvements [71]. However, it is important to note that neither of these trials was designed to measure efficacy, suggesting only the promise of disease treatment.

## 5. Induced Pluripotent Stem Cells in ALS Treatment

**Figure 4:**
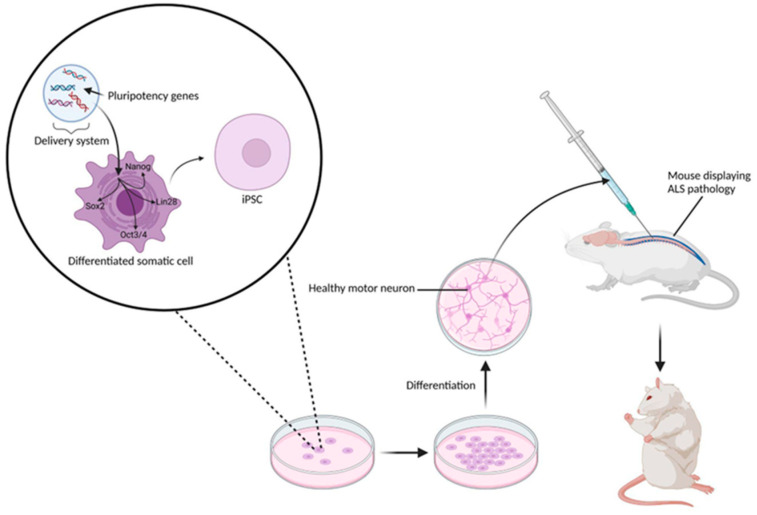
Typical preclinical experimental protocol involving the transplantation of iPSC-derived NSCs. The figure illustrates the delivery of pluripotency genes and the expression of pluripotency factors to induce pluripotency in differentiated somatic cells. The delivery system may be an integrating or non-integrating viral vector or a non-viral vector [76]. The figure also shows the Thomson factors being expressed; however, these specific pluripotency factors can be used interchangeably with numerous others. The iPSCs are cultured in a cell culture dish and differentiated into healthy motor neurons before subsequent intrathecal injection into a mouse with ALS disease phenotype. The produced outcome is a mouse with reduced symptoms. This figure was created using BioRender.

iPSCs, derived from patient-specific somatic cells, present the possibility of personalised therapy for ALS. iPSCs exhibit the unique ability for unlimited self-renewal and differentiation into all specialised cell types, offering a novel strategy by potentially replacing or supporting neurons and modifying the ALS-diseased microenvironment [74]. Pioneered in 2006 by Shinya Yamanaka and Kazutoshi Takahashi, the mechanism of pluripotency induction in iPSCs generally involves the exogenous delivery of pluripotency genes or factors (Figure 4) [33]. Pluripotency factors affect the methylation landscape of the epigenome by hypermethylating or hypomethylating specific DNA and histone regions [75,76]. Common pluripotency factors utilised in pluripotent stem cell (PSC) induction are the Yamanaka factors (Oct3/4, Sox2, Klf4, and c-Myc) and the Thomson factors (Oct4, Sox2, Nanog, and Lin28), which confer hypermethylation and hypomethylation, respectively [75]. Pluripotency factor genes are activated through demethylation of their promoter regions and are associated with increased H3K4 trimethylation [75,76]. These factors reprogram somatic cells into a pluripotent state similar to embryonic stem cells, allowing differentiation into any cell type [77]. Therefore, iPSCs are crucial for bypassing ethical issues related to embryonic stem cell use and enabling the production of patient-specific stem cells, lowering the risk of immune rejection in treatments.

iPSCs have the potential to treat ALS due to their ability to indefinitely duplicate and differentiate into nervous system cells, which is crucial for replenishing healthy motor neurons in ALS patients [75,78,79,80,81]. This characteristic underpins stem cell transplantation, the most common stem cell therapy for neurodegenerative disorders like ALS, where CNS repair is challenging due to the terminal differentiation of CNS cells. Loss of CNS connectivity, particularly the axonal connections between muscle and lower motor neurons via neuromuscular junctions, is central to ALS pathology [82]. Previous research by Kubo et al. (2009) demonstrated the ability of ESC-derived motor neurons to form new neuromuscular junctions. Given the close resemblance between iPSCs and ESCs, iPSC-derived motor neurons are anticipated to offer similar regenerative benefits [83]. Furthermore, paracrine signalling through extracellular vesicles from iPSC-derived neurons could provide additional therapeutic advantages by delivering neurotrophic factors and anti-inflammatory agents, thus promoting neuronal survival and aiding tissue regeneration [84]. This approach may be vital for re-establishing neuromuscular junctions, improving motor control in ALS, and modulating the CNS microenvironment to support neural repair.

Complementing this, Yang et al. (2023) developed a protocol to direct iPSCs into cervical spinal motor neurons (sMNs), representing a significant advancement in ALS research [85]. This method overcomes the complexities of embryoid body formation, yielding nearly pure NSCs. The hyperexcitability exhibited by these sMNs, a key phenotype of sporadic ALS, highlights their potential in disease phenotyping and modelling [85]. Another investigation into iPSC-derived neural progenitor cells (iNPCs) engineered to secrete GDNF underscores iPSCs’ neuroprotective capabilities in ALS [86]. The similarity in gene expression profiles of iNPCs to fetal neural progenitor cells and their demonstrated protective effects in rodent models of ALS further validate this therapeutic pathway. Marshall et al. (2023) extended iPSC applications by exploring axonal regeneration in hiPSC-MNs, particularly with the SOD1A4V mutation [87]. Their findings of enhanced axonal regeneration indicate the potential of IPSCs to counteract specific ALS deficits, such as impaired axonal regeneration, showcasing its targeted therapeutic strategy for ALS.

**Figure 5:**
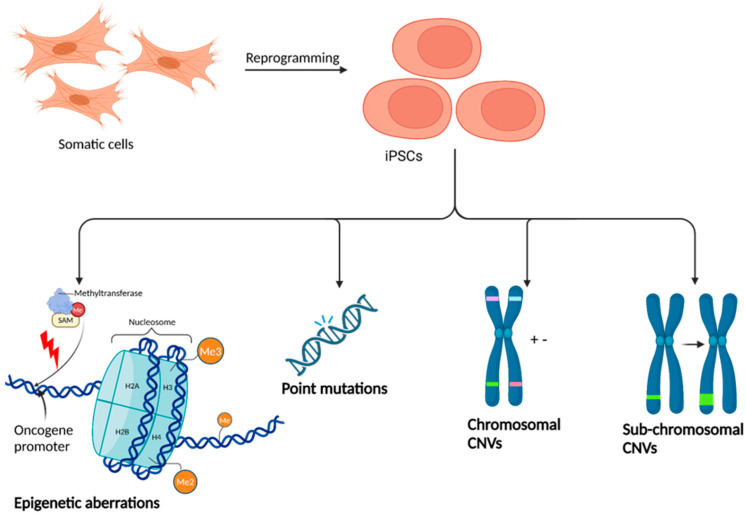
Potential genetic and epigenetic alterations caused by PSC induction. Epigenetic aberrations are shown through the illustrated interference of promoter methylation in the major groove of an oncogene segment within a typical chromatin landscape. Methyltransferase is associated with S-adenosylmethionine (SAM), which functions as a methyl donor. Point mutations are displayed through the breakage of the DNA backbone and the subsequent absence of deoxynucleotide base pairs. CNVs are shown where chromosomal segments are either duplicated (+) or deleted (−). Sub-chromosomal CNVs, which involve smaller segments within the chromosomes, are also presented. These genetic changes can have significant implications for using iPSCs in clinical settings, necessitating rigorous screening and validation. This figure was made using BioRender.

The safety profile of iPSC-derived cells in ALS therapy is generally considered favourable, though concerns about genotoxicity and epigenetic irregularities persist (Figure 5) [75,78,79,80,81]. Research suggests that genomic alterations in iPSCs, while typically infrequent and parentally inherited, can include de novo SNVs as well as chromosomal and sub-chromosomal copy number variations (Figure 5) [76,88]. Notably, these mutations, when affecting coding regions, are often concentrated within proto-oncogenes [75,76]. A strategic approach to mitigate these risks involves replacing c-Myc with Lin28 in the reprogramming factor cocktail, enhancing cellular resilience to DNA damage and stress, and potentially reducing epigenetic aberrations similar to those present in tumorous cells [89]. A primary consideration is also the delivery method of pluripotency factors. Though viral vectors are the most traditional method of PSC induction, they tend to produce stronger immune responses and or larger amounts of genetic and epigenetic abnormalities [75,76]. Delivery using modified mRNAs solves these issues by providing a nascent approach to safely and cost-effectively generating iPSCs [76]. Clustered regularly interspaced palindromic repeats (CRISPR) technology also offers a relatively safe and cost-effective solution to targeting specific genes for efficient generation of iPSCs [76]. Future research in stem cell induction should focus on modified mRNA and CRISPR delivery systems. Most studies indicate iPSC therapy is safe, primarily because iPSCs are derived from the patient’s somatic cells, thereby reducing the risk of immune rejection [75,78,79,80,81]. Preclinical trials have shown the safety and efficacy of transplanting iPSC-derived NSCs (iNSCs), but more human research is needed [90,91,92]. Current studies on iPSC-derived NSC transplantation in ALS have been underpowered, highlighting the necessity for more comprehensive research, including randomised control trials. These trials should ideally compare phenotypic biomarkers and physical mobility outcomes between patients receiving transplants of iNSCs, BMSC-derived NSCs, ESC-derived NSCs, and controls. This comprehensive approach to evaluating iPSC safety and efficacy may be essential for validating their use as a viable therapeutic option in ALS and other conditions.

iPSC therapy, while promising for treating diseases like ALS, faces significant limitations, particularly regarding its high cost [93]. One critical advantage of iPSCs is the ability to self-renew, which theoretically allows for the generation of extensive cell banks [93]. This self-renewal property is fundamental to therapy involving iPSCs, enabling the continuous production of specialised cell types. However, establishing and maintaining these cell banks requires meticulous quality control, standardised procedures, and rigorous characterisation, all of which contribute to the high costs associated with iPSC therapy. These costs are compounded by the need for thorough testing of sterility, cell phenotype, chromosomal stability, genetic identity, and pluripotent potential, ensuring the safety and efficacy of the iPSCs used in therapies. Furthermore, ethical considerations like informed consent from both cell donors and recipients, as well as protecting personal genetic information, are paramount in iPSC therapy [93]. All procedures, from cell collection to iPSC application, must adhere to good manufacturing practice standards. The integrity of methods to derive or differentiate iPSCs, especially those involving genetic manipulation, is crucial. The potential misuse of iPSCs for human cloning, creating human–animal chimaeras, or illicitly generating modified human gametes calls for ongoing ethical debate and regulatory vigilance.

## 6. Future Directions of Stem Cells

In this review of current stem cell applications in ALS treatment, we have identified the key distinct therapeutic potentials and challenges associated with each cell type [Table 4]. The rapid disease progression of ALS, paired with its low incidence, has contributed to the low sample size in clinical trials of pre-symptomatic or early symptomatic populations. The majority of symptomatic ALS cases are in the advanced stages. Rapid disease progression and low incidence of ALS have resulted in underpowered clinical trials focusing on pre-symptomatic or early-symptomatic individuals. The outcome is limited temporal analysis of neurological tissue except in post-mortem. This reflects the challenges of ALS heterogeneity in investigations’ methodologies where detailed comparisons between differing stem cell types and studies are lacking. Cohorts are generally from similar backgrounds in developed countries, which does not consider the impact of demographics on the accessibility of treatment and surveillance of ALS. Moreover, the ethical issues arising with human-derived stem cells exacerbate the complexities in realising a long-term therapy that is effective and reliable.

### 6.1. Future Applications of Mesenchymal Stem Cells

MSCs, obtained from bone marrow, adipose tissue, or umbilical cord, are noted for their immunomodulatory and regenerative capabilities. MSC clinical trials have reported slowed disease progression and improved survival, especially with intrathecal BMD-MSC administration. Despite these promising results, challenges of variability in MSC properties, inconsistent clinical outcomes, and insufficient long-term safety data persist [Table 4]. MSCs exhibit notable immunomodulatory properties, as demonstrated in clinical studies of intrathecal administration with BMD-MSC. These studies suggest a potential deceleration in ALS progression for certain patients. However, MSC research faces methodological constraints using non-randomised study designs and small sample sizes, hindering the ability to definitively conclude the effectiveness of MSCs in treating ALS. The varying methods in MSC preparation and administration across studies complicate the evaluation of result consistency, with the majority being trials on animal models. Phase III clinical trials, including Cudkowicz et al. [46] and Nam et al. (2022), attempt to resolve the aforementioned weaknesses through randomised control trials, larger sample sizes, and control groups [56]. Additional phase III trials need to be conducted to reliably compare the efficacy of MSCs derived from different sources to determine the most effective stem cell type for ALS treatment, which could be accomplished through standardising research administration and preparation. Phase IV clinical trials monitoring the long-term effects and data collection of MSC therapy in diverse patient populations should be conducted once there is a comprehensive agreement on the safety and efficacy proven in phase III trials. Cross-comparative trials compare various sources of MSCs in the same trial to directly assess their relative effectiveness and safety profiles, alongside advanced biomarker analysis to better understand the mechanisms of action of MSCs and identify predictors of response to combination therapy.

### 6.2. Future Applications of Neural Stem Cells

NSCs, derived from the fetal spinal cord or brain, show promise in differentiation into neurons and glia, offering neuroprotection and a potential slowing of disease progression. These cells have demonstrated delayed motor dysfunction and extended survival in rodent models and some improvements in functional scores in human trials [Table 4]. However, due to the preliminary nature of the trials, their use is limited by ethical concerns, invasive procedures, limited cell migration and survival post-transplantation, and inconclusive efficacy. There is also limited research after 2020 as attention turned to iNSCs to counteract ethical concerns of embryonic stem cells, which are derived from aborted fetuses, while achieving larger sample sizes. ALS’s rapid and progressive nature means the long-term effects of NSC transplantation are not well known. It can only be inferred using post-mortem tissue, as stated by Baloh et al. (2022), where 13 deceased patients’ tissues were used to demonstrate safety and graft survival [72]. Prior studies had low-minimal migration of NSCs away from the grafted site with minimal production of NCP-derived neurons and circuit reassembly occurring [97,98]. Modified NSCs containing GDNF aimed to address this by improving NSC survival length [72]. Baloh et al. (2022) noted benign neuromas as a localised proliferation of Schwann cells around the infection site not detected by MRI [72]. These were likely caused by damage to the injection site and dorsal root entry zone. Future studies should aim to avoid the aforementioned site while targeting deeper into the ventral horn. NSC survival and migration could also be further enhanced post-transplantation through genetic modifications or co-administration with supportive factors such as GDNF. Animal trials for efficacy face problems of genetic background variability in mix-strained mice. Despite the significant promise in preclinical SOD1G93A ALS models, their limited ability to proliferate and differentiate has meant that beyond the safety of the administration of NSCs, any therapeutic benefits are not apparent. Controls of sex, age, and quantifiable measures of disease progression (early symptomatic only) were not considered [72]. Therefore, translating positive results from animal studies to human clinical trials poses considerable challenges that require further investigation, meaning it will be necessary to develop reliable biomarkers to monitor NSC engraftment, survival, and functional integration. Additionally, most clinical trials from clinicaltrials.gov do not include other databases, such as the EU Clinical Trials Register and the Japan Registry for Clinical Trials [87]. It may exclude other backgrounds, as Wei et al. (2019) mention an association between ALS genetic susceptibility and population origin [99]. Ghosh et al. (2022) further comment that underrepresented groups produce disparities in polygenic risk scores [100]. NSC’s capability to effectively treat ALS sub-variants known to occur in specific demographics is likely overestimated [101]. Therefore, larger randomised controlled trials incorporating diverse patient populations can identify the long-term efficacy of NSC therapy, while the integration of NSCs with other therapeutic modalities and gene editing tools such as CRISPR may enhance treatment outcomes.

### 6.3. Future Applications of Induced Pluripotent Stem Cells

Potentially resolving the ethical and differentiation limitations of MSCs and NSCs, iPSCs are noteworthy for their unlimited self-renewal and capability for a highly valuable personalised therapeutic approach in ALS [102]. iPSCs, derived from patient-specific somatic cells, stand out for their unlimited self-renewal and differentiation into specialised cell types. They have shown potential in rodent models for improving motor function and reducing glial activation. The technical complexity of iPSC generation, high costs, risk of tumorigenicity, and the need for controlled clinical trials to establish efficacy and safety profiles remain significant hurdles [Table 4]. While iPSCs can potentially replenish degenerated motor neurons, presenting a novel strategy for disease intervention, they also face challenges related to genotoxicity and epigenetic abnormalities, alongside high production and application costs. Hence, this presents a significant barrier to their widespread use in clinical settings.

Research by Vogt et al. (2018) indicates that TDP-43 can induce p53-dependent apoptosis of immature cortical neurons derived from human iPSCs. TDP-43 variants are also able to inhibit p53, thereby preventing IPSCS-induced death of human cortical neurons in ALS patients [103]. However, the study notes that reducing TDP-43 can lead to defects in NSC proliferation during neurodevelopment. Thus, its potential risks need to be further evaluated. Moreover, CRISPR technology can induce pluripotency factors in patient motor neurons to avoid rejection of host grafts from donor iPSCs [102]. However, the off-target effects of the CRISPR system require careful management to mitigate additional mutations in iPSCs. Bioinformatic tools such as TIDE, CRISPR-GA, Cas-Analyzer, and CRISPResso play a crucial role in identifying potential off-target sites [104]. The quality control reads quantify the editing efficiency and occurrences of repair pathways non-homologous end joining (NHEJ) and homology-directed repair (HDR) in the genome, thus aiding in the design of more precise CRISPR-Cas9 interventions [104]. Alongside this, alternative techniques are being investigated to further minimise mutation risks. These include the use of specific Thomson factors as opposed to Yamanaka factors, the strategic employment of guide RNAs, the application of Cas9 nickases, which induce single-strand breaks rather than double-strand, and the incorporation of anti-CRISPR proteins [105]. Each of these approaches enhances the specificity and safety of gene editing in iPSCs. Furthermore, establishing comprehensive databases, such as Answer ALS, is pivotal for generating clinical and biological signatures to elucidate ALS’s underlying mechanisms, including subgroup identification [106].

### 6.4. The Prospect of Combination Therapy

Stem cell combination therapy has emerged as a promising pathway involving the transplantation of two or more different cell types to increase synergistic effects and efficacy of treatment. Although the research area is recent, clinical trials for neurodegenerative diseases have reported improved engraftment and regenerative potential, overcoming the disadvantages of isolated stem cell therapies. Duchenne muscular dystrophy is a progressive muscle degeneration disease that affects the CNS and causes developmental impairment and seizures [107]. In a study by Klimczak et al. (2020), increases in motor function and delayed disease progression through the co-transplantation of BM-MSCs and skeletal muscle-derived progenitor cells were noted [108]. Co-transplantation of differing stem cells has also been studied in other neurodegenerative diseases such as spinal cord injury, ischemic stroke, and retinal degenerative diseases, with results suggesting long-term safety, increased neuronal differentiation, and survival rate [109,110,111]. Although only combined stem-cell-gene therapy has been studied for ALS, stem-cell combination therapy shows promise in promoting growth and migration for many neurological disorders, with future research required to evaluate its application within the complexity of ALS [72].

## 7. Conclusions

This review aimed to assess the potential of stem cell therapy for treating ALS, focusing on the therapeutic applications of MSCs, NSCs, and iPSCs and evaluating their benefits and limitations. The main findings indicate that each stem cell type offers distinct therapeutic potential for ALS treatment. MSCs have demonstrated potential for slowing the progression of ALS and improving patient survival. Their immunomodulatory and regenerative properties have shown promise in both preclinical and clinical trials. NSCs can differentiate into neurons and glia, offering neuroprotection and the potential to slow ALS progression. Clinical trials with NSCs have generally reported positive safety outcomes, and some studies noted functional improvements in ALS patients. iPSCs can be derived from patient-specific somatic cells, allowing personalised therapy and the possibility of replacing or supporting neurons in ALS patients. These cells offer unlimited self-renewal and have shown promise in preclinical studies with positive results in rodent models. However, MSCs face limitations due to inconsistencies in clinical outcomes, non-randomised trial designs, and variable MSC properties based on their source, complicating their standardisation and efficacy. NSCs are hindered by ethical concerns surrounding fetal tissue, the invasive nature of intraspinal delivery, and limited cell migration, challenging their broader application in treating ALS. iPSCs encounter high costs, risks of tumorigenicity or genotoxicity, and complex generation techniques, which require comprehensive controlled trials to ensure safety and efficacy in ALS therapy. Stem cell therapy offers a revolutionary multi-target approach to ALS treatment that may provide a primary or adjunctive therapy with improved efficacy in comparison to current treatments.

## Figures and Tables

**Table 2:** Summary of NSC SOD1G93A rat clinical trials.

Subject	Cell Type	Delivery	Dosage	Experimental Protocol	Reported Results	Reference
G93A-SOD1 mice (70 days old)	Extracted NSCs	Lateral ventricle and tail vein	5 × 10^5^ (lateral ventricle) 1 × 10^6^ (tail vein)	Total of 78 mice tested (including control group)	Lateral ventricle pathway preferential. Limited effects NSC to protect the anterior horn motor neurons and increase survival time.	[66]
Transgenic 1 G93A transgenic rats (early symptomatic)	hNSCs isolated from from fetal brain	Four bilateral transplantations into the anterior horns of L3–L4	15 days of immunosuppression with 4 × 10^5^ cells intraspinal	Controls of untreated SOD1 rats and rats treated with HBSS Abercrombie formula used to determine hNSC quantity for graft region	Survival rate increased by up to 38%. Improved rotarod test. Extensive migration (3.77 ± 0.63 cm) of hNSC from injection site	[64]
Mutant SOD1G93 mice (mice 8–10 weeks old)	hNSC from the telencephalic ventricular zone of 13 weeks fetus	Injections of hNSCs bilaterally at L2 or C6, T10, L1, and L3	Single injection of 10^5^ cells first site, 10^5^ cells second site, 10^5^ cells third site	11 independent, double-blinded studies	Approximately 20–40% survival rate increase of 200–365 days. Site 3 > Site 2 > Site 1 improved hind leg movement. Found no improvement when NSCs were injected in late symptomatic mice.	[62]
SOD G93A rats (63 days old)	Cervical–thoracic cord of a single 8-week fetus	Both sides of ventral horn of L4–L5 and C4–C5	2 × 10^4^ cells/1 μL per injection site, total 2.4 × 10^5^ cells per animal from 12 injections	Motor strength tests included the BBB rating	Approximately 68.8 ± 0.07% of surviving C4–C5 NSCs differentiated into neurons. Extended survival of 17 days.	[65]

**Table 3:** Summary of human clinical trials centred on intraspinal hNSC safety.

Phase	Enrolment	Delivery	Dosage	Experimental Protocol	Reported Results	Reference
I	12	Five unilateral or five bilateral (ten total) in lumbar spinal cord	100,000 cells/injection	Non-ambulatory Group A. Ambulatory Groups B and C. Assessments (6–18 months).	No reported long-term complications. One patient demonstrated improvement in disease progression (trial not designed to measure efficacy).	[68,73]
I	18	Microinjections intraspinal of hNSCs into the grey matter of spine Group 1: lumbar [T8/11], Group 2 and 3: cervical [C3/5]	Group 1: 2.25 × 10^6^ and 4.5 × 10^6^ Group 2: 2.25 × 10^6^ and 4.5 × 10^6^ Group 3: 4.5 × 10^6^	Three cohorts of six patients each (aged 20–75) Group 1: not-ambulatory Group 2 and 3: ambulatory All groups: FVC ≥ 60% of predicted value	Safety up to 60 months. Up to 4 months after intervention, ALSFRS-R “detected a transitory decrease in progression”. Two improved ambulation scores. Eleven deaths, two tracheostomies.	[71]
II	15	All groups: bilateral injections at the C3 through C5 cervical segments. Group E: L2 through L5 lumbar segments.	Dose escalation: 10 μL per injection separated by 4 mm.	Five sequential cohorts (each with 3 patients)—Groups A–E. Ambulatory Groups (D + E). Group E same as Group C in Glass et al. (2012) reported trial. Only historical control groups.	No identified acceleration of disease progression. Improved ALSFRS-R and ALS/SURV scores after 24 months. Two deaths due to natural disease progression.	[69,70]
I/IIa	18	Unilaterally to the lumbar region	Two escalating doses	Single-centre, blinded (concerning injection side)	No negative effect on motor function up to 42 months post-transplantation. Occurrence of benign neuromas close to injection site.	[72]

**Table 4:** Comparative analysis of stem cell therapies in neurodegenerative disease research.

Stem Cell Type	Source	Study Phase	Therapeutic Effects	Study Outcomes	Limitations and Considerations	References
MSCs	Bone marrow, adipose tissue, umbilical cord	Preclinical and Phase I/II/III clinical trials	Immunomodulation, neuroprotection, regeneration, potential slowing of disease progression.	Some clinical trials reported slowed progression and improved survival probabilities, particularly with intrathecal BMD-MSC administration. Preclinical studies in animal models showed delayed motor dysfunction and neuroprotective effects.	Variability in MSC properties based on source tissue, inconsistency in clinical outcomes, non-randomised and uncontrolled study designs, retrospective analyses that limit causal inferences, and insufficient long-term safety and efficacy data.	[45,53,56,58]
NSCs	Fetal spinal cord or brain	Preclinical and Phase I/II clinical trials	Differentiation into neurons and glia, neuroprotection, potential slowing of disease progression.	Delayed motor dysfunction and extended survival in rodent models. In human trials, some improvements in functional scores were observed, and the procedures were generally considered safe.	Ethical concerns with fetal sources, invasive procedures for intraspinal delivery, limited migration and long-term survival of grafted cells, lack of conclusive evidence for efficacy due to small, non-randomised trials with short follow-up periods.	[62,63,64,65,66,68,69,70,71,72,73]
iPSCs	Patient-specific somatic cells	Preclinical trials	Unlimited self-renewal, differentiation into specialised cell types, potential replacement of neurons, disease modelling.	Rodent models have shown improved motor function, preservation of motor neurons, and reduced glial activation with iPSC-derived neuron transplantation. Potential in personalised disease modelling and drug screening.	Technical complexity of iPSC generation and differentiation, high costs of production and maintenance, risk of tumorigenicity and genomic instability, limited human trial data, and a significant need for controlled clinical trials to establish efficacy and safety profiles.	[74,75,76,78,79,80,81,83,84,88,94,95,96]

## Data Availability

No new data were created or analyzed in this study. Data sharing is not applicable to this article.

## Abbreviations

ALS	Amyotrophic lateral sclerosis
ALSFRS-R	ALS functional rating scale-revised
ASC	Adipose-derived stem cells
BBB	Basso, Beattie, Bresnahan (motor strength test)
BDNF	Brain-derived neurotrophic factor
BMSCs/BMD-MSCs	Bone marrow-derived mesenchymal stem cells
BMD-MNCs	Bone marrow-derived mononuclear cells
Cas9	CRISPR-associated protein 9
c-Myc	Cellular myelocytomatosis proto-oncogene
CNS	Central nervous system
CRISPR	Clustered regularly interspaced palindromic repeats
CRISPR-Cas9	CRISPR-associated system for gene editing
CRISPR-GA	CRISPR guide alignment
CRISPResso	CRISPR analysis tool for editing efficiency
FVC	Forced vital capacity
FDA	Food and Drug Administration
ESC	Embryonic stem cells
GABAA	Gamma-aminobutyric acid type A
GDNF	Glial cell-derived neurotrophic factor
GFAP	Glial fibrillary acidic protein
HDR	Homology-directed repair
hNSC	Human neural stem cells
iNPCs	Induced neural progenitor cells
iNSCs	Induced neural stem cells
iPSCs	Induced pluripotent stem cells
MCP-1	Monocyte chemoattractant protein 1
MRI	Magnetic resonance imaging
mRNA	Messenger RNA
NfL	Neurofilament light
NHEJ	Non-homologous end joining
NSCs	Neural stem cells
PCR	Polymerase chain reaction
PSC	Pluripotent stem cell
p53	Tumour protein p53
qNSC	Quiescent neural stem cells
SAM	S-adenosylmethionine
SICI	Short-interval intracortical inhibition
SCs	Stem cells
sMNs	Spinal motor neurons
SNVs	Single nucleotide variants
SOD1	Superoxide dismutase 1
SOD1A4V	SOD1 gene mutation (A4V)
TDP-43	TAR DNA-binding protein 43
TGF-β1	Transforming growth factor beta 1
TUJ1	Neuron-specific class III beta-tubulin
UC-MSCs	Umbilical cord-derived mesenchymal stem cells
UCMSC-CM	Umbilical cord mesenchymal stem cell-derived conditioned medium
WBC	White blood cells
WJ-MSCs	Wharton’s jelly mesenchymal stem cells
